# Medical Society of the County of Erie

**Published:** 1885-07

**Authors:** Edward Clark

**Affiliations:** Secretary


### Medical Society of the County of Erie.

*Semi-Annual Meeting, June 9th, 1885.*

The semi-annual meeting of the Medical Society of the County of Erie was held at the Y. M. C. A. Building on Mohawk street Tuesday, June 9th, 1885. The meeting was called to order at 10.30 A. M. by the President of the Society, Dr. J. B. Andrews, and the following members were present, viz.: Drs. Cronyn, F. F. Hoyer, Pryor, VanPeyma, Persons, Willoughby, G. W. McPherson, Johnson, Barnes, Haberstro, Wetmore, J. H. Potter, Wm. G. Ring, Wm. Ring, W. D. Bidaman, F. W. Abbott, Gay, F. H. Potter, S. H. Warren, J. W. Putnam, Dorland, W. W. Potter, B. P. Hoyer, Hubbell, Croakley, Briggs, Gumaer, B. G. Long, Bailey, Frederick, Mary Berks, Armstrong, Keene, Sheehan, Walsh, Brecht, Rochester, Howe, O'Brian, Ballou, Vandenberg, Gregory, Ellwood, Bissell, Packwood, Barker, Wilson, Samo, Runner, Hayd, Mullen, E. H. Long, Crawford, Daggett,

Greene, Harvey, E. E. Storck, Bartlett, Jewett, Kamerling, York, Hauenstein, Howell, Phelps, Niemand, E. Storck, and by invitation Drs. Stanley, Bode, Whitnall, Rich, and De Lancey, Rochester.

The minutes of the annual meeting of January 13th were read and approved. Drs. Vandenberg and Wm. G. Ring, who were elected at the last annual meeting, were introduced to the Society.

The Committee on Membership reported as follows :

"Your committee on membership would respectfully report that they find that the diplomas of Drs. C. F. Howard and Thos. G. Sheehan were granted by medical schools in good and regular standing. The diplomas of Dr. D. W. C. Greene and Dr. J. E. Johnstone were not presented for examination to the committee on membership, so that action on the names of these two gentlemen will have to be deferred until the next regular meeting." The report of the Committee on Membership was unanimously adopted, notwithstanding an affidavit presented by a member of the Society, was read, which set forth the fact that Dr. Howard practised homeopathy, etc.

Dr. Storck, chairman of the Board of Censors, reported that since the last regular meeting the Board had succeeded in having four irregular practitioners arrested. One, a so-called Prof. Ginner, whose headquarters were at the Tifft House, and who claims to have graduated at the Belleville College of Boston, Mass. Another, a Polish doctor, who, after spending one night in jail, left the city. Against the other two, viz. : a so-called Prof. Hartley and a Doctor Brown, the Board failed to make out a case, for at their trial it was shown that "Prof. Hartley" was not practising medicine and that Dr. Brown claimed to have graduated at a "Homeopathic College" in good standing in New York, producing a diploma to that effect, consequently they were both discharged.

In conclusion, Dr. Storck recommended that an appropriation of $25.00 be made to pay the expenses incurred by the board.

On motion of Dr. Dorland the report was received and recommendations contained therein adopted.

Dr. Johnston moved that a vote of thanks be tendered to the Board of Censors, which was carried.

The committee appointed to revise by-laws reported progress and asked for further time, which was granted.

The Committee on Legislation made no report.

Under the head of "Miscellaneous Business," the secretary submitted a draft of a new membership certificate; and, on motion of Dr. W. W. Potter, a committee of three was appointed, with power to procure 500 copies of a new certificate, and to report at the next regular meeting. The President appointed, as such committee, Drs. Clark, W. W. Potter and T. W. Abbott.

Under this head, Dr. Dorland offered the following resolution, which was unanimously adopted:

*Resolved,* "That the Secretary notify every applicant for membership to present himself and his diploma to the Chairman of the Committee on Membership, prior to the next regular meeting of the Society, following the presentation of his application."

Under the head of "Appointment of Essayist," Dr. Howe moved that a committee of three be appointed, with power to regulate and control the presentation and reading of all essays, papers, etc., hereafter to be presented for the Society's consideration. Carried. The President appointed Drs. Howe, Briggs and Putnam as such committee.

There being no other business before the meeting, the Society adjourned.

EDWARD CLARK, M. D., Secretary.

